# Building and Sustaining Community Engagement to Advance School Behavioral Health Research

**DOI:** 10.3390/bs15081080

**Published:** 2025-08-08

**Authors:** Kristen Figas, Katherine A. Perkins, Brian P. Daly, Robert Stevens, Brooke E. Chehoski, Mark D. Weist

**Affiliations:** 1 Department of Psychology, University of South Carolina, Columbia, SC 29208, USAkp40@mailbox.sc.edu (K.A.P.);; 2 Department of Psychological and Brain Sciences, Drexel University, Philadelphia, PA 19104, USA; 3 Southeastern School Behavioral Health Community, Columbia, SC 29208, USA

**Keywords:** community engagement, school behavioral health, interconnecting research and practice

## Abstract

The promise of achieving desired outcomes in community-engaged research relies upon an ongoing and long-term connection between the community and researchers. However, many community–researcher relationships begin and end in the confines of a single project, often precluding the sustainability and scalability of programs and initiatives that can benefit the community. Few examples exist in the literature, especially for the focus of this paper—school behavioral health (SBH)—to understand how the complex, challenging, and nuanced process of continued engagement between researchers and community members can be sustained and succeed. In this article, we chronicle the development of the Southeastern School Behavioral Health Community across 13 years, from its inception in a single state to its regional expansion through two research awards, to illustrate how long-term community engagement and a history of community connections can shape SBH research and practice across project action cycles. We describe the strengths, challenges, and lessons learned from this long-term community engagement experience. Numerous examples illustrate proactive and responsive strategies for initiating and sustaining community engagement throughout all phases of the longitudinal initiative and demonstrate tangible ways in which meaningful engagement influenced both research and practice. The reflections include the extent to which engagement principles of the research funder (the Patient-Centered Outcomes Research Institute, PCORI) were enacted during this research program; our roles as researchers, facilitators, and community members; the impact of the COVID-19 pandemic; engagement facilitators and structures; and what was achieved regarding levels of engagement. Future directions are provided for sustaining interconnected, community-engaged research and practice in SBH.

## 1. Introduction

Child and youth behavioral health is a critical concern for the present and future thriving of communities around the globe. The development and improvement of methods for tracking child and youth behavioral health have provided researchers, communities, and policy leaders with opportunities to analyze critical trends within and across geographies (Whitney & Peterson, 2019), racial groups (Sheftall et al., 2022), sexual minority populations (Lucassen et al., 2017), and refugees (Kien et al., 2019) and in the context of stressful experiences associated with the COVID-19 pandemic (Figas et al., 2023; Samji et al., 2022). In the context of the increasing prevalence rates of mental health challenges among children and youth, the U.S. Surgeon General (2021) declared a youth mental health crisis and made an urgent call to increase innovation to address this crisis, including increasing mental health support in schools.

Most behavioral health difficulties develop during childhood, adolescence, and early adulthood (Kessler et al., 2007). Access to care remains a persistent challenge, especially for underserved and disenfranchised populations (Mongelli et al., 2020). Fortunately, universal behavioral health promotion and high-quality selective and intensive early intervention efforts can reduce the severity and duration of behavioral health challenges, prevent secondary health conditions from developing, and intercept subsequent periods of recurring difficulty (de Girolamo et al., 2012). For youth, these efforts are often delivered through expanded school mental health programs, which grew significantly in the U.S. in the 1980s and 1990s, integrating community mental health providers to expand mental health services into schools (Short & Talley, 1997; Weist, 1997). The availability of school-based services resulted in higher service utilization rates and acceptability (So et al., 2019), to the point where schools are now the most prominent sites for mental health intervention for children and youth (Duong et al., 2021; Evans et al., 2023).

School behavioral health (SBH) programs emphasize education–mental health system integration, with collaborating community-based clinicians moving into schools to augment the work of school employees (e.g., school psychologists, counselors, social workers, nurses) to build a full continuum of Tier 1 prevention, Tier 2 early intervention, and Tier 3 treatment services, which are associated with the multi-tiered system of support (MTSS) framework increasingly adopted by schools (Eber et al., 2020; Weist et al., 2020). These services are being advanced to reduce stigma; improve the depth, quality, and effectiveness of child and youth behavioral health services; enhance cultural responsivity; and identify and reduce disparities (Weist & Christodulu, 2000). Community engagement is imperative for implementing SBH so that it is appropriately adapted and adhered to and achieves the desired outcomes. Therefore, SBH must be accessible, acceptable, appropriate, feasible, and sustainable in every context, and community engagement is a key process, if not the key process, for supporting these outcomes.

### 1.1. Community Engagement in Research

Community engagement in research has been prioritized for decades (see Collins et al., 2018). In general, however, engaging with communities has frequently been perceived as optional by researchers. However, leading researchers have recently called on research funders and research institutions to make community engagement obligatory and not optional in the research process (Grumbach et al., 2021). Although many outcome studies of community-engaged research exist in the current literature, few studies detail specific action steps for how an initial project grant can develop into a sustainable partnership. For future community engagement efforts to truly be embedded in the research enterprise, examples are needed for researchers and community members to learn about best practices and successful approaches to sustaining these relationships. The Patient-Centered Outcomes Research Institute (PCORI, see www.pcori.org (accessed on 29 July 2025)) funds healthcare research that involves community partners and patients as essential collaborators in the research enterprise. PCORI is an independent nonprofit research organization created through the 2010 Patient Protection and Affordable Care Act that emphasizes community engagement through all stages of the research process (Frey et al., 2017). PCORI requires community engagement when planning a study, conducting the study, and disseminating the results, and community engagement follows six principles: reciprocal relationships, co-learning, partnerships, transparency, honesty, and trust (PCORI, 2014). PCORI recently expanded upon these principles, specifying six foundational expectations for partnerships in research: diversity and representation, early and ongoing engagement, dedicated funds, building capacity to work as a team, meaningful inclusion of partners in decision making, and ongoing review and assessment of engagement (PCORI, 2024).

In this paper, we describe two research studies funded by PCORI: an early, smaller Eugene Washington Engagement Award (2015–2017, EAIN-2874), which led to a large-scale comparative effectiveness trial (2019–2025, IHS-2018C1-10928). We analyze community engagement for both projects through a framework proposed by Key et al. (2019), which is a seven-category continuum of community-engaged research, from no community involvement to community-led research, with community-based participatory research (CBPR) falling closest to community-led work. Along this continuum are seven indicators of equity (power and control, decision making, influence, mutual benefit, ownership, responsibility, and resource sharing) and five contextual factors (history, trust, relationship building, respect, and transparency). This framework was used as it aligns with PCORI’s original principles and key contextual factors, as well as the newest expectations that address dimensions of equity.

### 1.2. Community-Engaged Project Design: Developing a School Behavioral Health Community

As stated above, the two PCORI-funded projects were developed in the context of a 13-year process to foster cross-sector community collaboration to advance SBH: first at a state level and then at a regional level. The South Carolina School Behavioral Health Community (SCSBHC) was established in 2012 and connected leaders from a variety of youth-serving systems (e.g., education, mental health, child welfare, juvenile justice, healthcare, family and youth advocacy) across all counties in South Carolina, holding its first interdisciplinary conference in 2014. The second conference in 2015 included a forum attended by diverse community members from across the state, including those listed above and youth and families, to identify areas of need and generate ideas for advancing SBH in South Carolina. Discussions at this forum generated five critical themes: building cross-sector partnerships, developing effective school-wide approaches for supporting students’ mental/behavioral health, promoting cultural responsiveness and humility, improving service quality through increased use of evidence-based practices (EBPs), and enhancing implementation support for EBPs (Weist & Stevens, 2017).

### 1.3. PCORI Engagement Award

**Figure 1:**
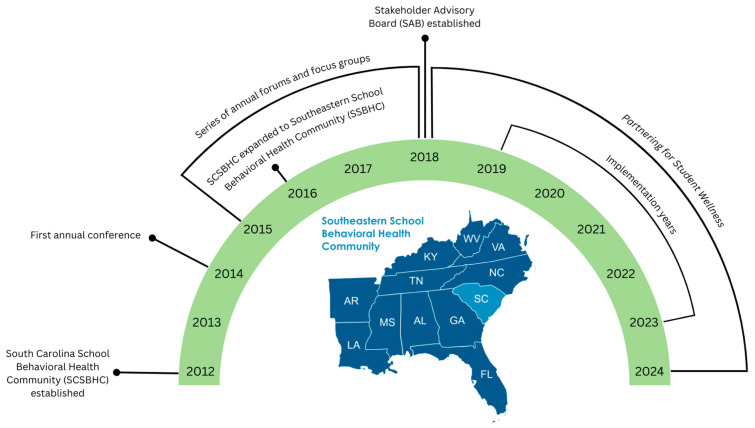
Geography of the SSBHC and timeline of key events.

Following the 2015 conference, SCSBHC leaders received the PCORI Engagement Award to advance SBH across the southeast region. This began with separate focus groups involving researchers, community partners, and behavioral healthcare recipients across South Carolina, focusing on the five core themes reviewed in the above section (see Weist et al., 2020). In addition, PCORI leaders and community partners from the Engagement Award recommended the expansion of the SCSBHC to become the Southeastern School Behavioral Health Community (SSBHC). To this day, the SSBHC connects leaders, practitioners, youth, and families across education, mental health, and other youth-serving systems throughout the southeastern region of the U.S. through a website (see www.schoolbehavioralhealth.org), a large and growing listserv, and an annual SBH conference (drawing over 1000 interdisciplinary participants in recent years). The geography of the SSBHC and a timeline of key events are depicted in Figure 1.

At the 2016 conference, as part of the Engagement Award, over 40 diverse community members attended a second forum to explore ways of capitalizing on findings emerging from the five focus groups. A third forum was held at the 2017 conference to distill recommendations. Based on forum feedback, additional focus groups were then conducted for three priority populations: children and youth in the child welfare system, youth connected to juvenile justice, and youth from military families. Ultimately, eight total focus groups were conducted, and data from them were qualitatively analyzed, yielding many ideas for advancing effective SBH overall and for each of the eight focus group topics (Weist et al., 2020).

The ideas generated from the research and community partners of the Engagement Award provided the impetus for a research project examining enhancements to SBH. A newly formed study team identified evidence-based approaches to addressing each theme, in partnership with select community members, using all community engagement data. A diverse sample of participants (representing school administrators, educators, school counselors, school-based mental health clinicians, psychologists, researchers, and community/family/patient advocacy organizations) completed a survey distributed to all attendees of the 2018 conference to refine and select approaches by rating 10 prospective intervention elements. Thus, iterative community engagement was critical to both the inception and development of a large comparative effectiveness trial (described below). Following a brief description of the trial, we illustrate the ways in which community engagement was sustained throughout the project, informing planning, implementation, and dissemination.

### 1.4. Comparative Effectiveness Trial—The Partnering for Student Wellness Project

The primary aim of the Partnering for Student Wellness (PSW) project was to evaluate the added impacts of research and community partner-recommended enhancements from the Engagement Award on the core strategies for effective SBH identified from prior research. From over 50 themes discerned from the eight focus groups, our analysis indicated that four themes were particularly prominent: (1) the pervasiveness of stigma as a barrier to SBH connection and hence a focus on mental health literacy; (2) the need for increased family leadership in SBH planning and implementation; (3) the critical importance of clinician–student relationships and focusing on therapeutic alliance; and (4) cultural responsiveness. In a comparative effectiveness trial conducted over four school years (2019–2020, 2020–2021, 2021–2022, and 2022–2023), 20 middle schools from a Southeastern and a Midatlantic state (10 each) participated, with all schools receiving training and implementation support for established evidence-based practices for effective SBH (modular cognitive behavioral therapy, promoting family engagement in care, and focusing on quality assessment and improvement) and with half of the schools (random selection of five from each site) receiving training and implementation support for the above four enhancements. While the focus of this paper is not on this comparative effectiveness trial, the findings revealed that students receiving the augmented intervention were more likely to receive telehealth intervention (instituted during the pandemic) and showed more improvements in emotional/behavioral functioning at 6-month post-assessment. There were also trends indicating that schools receiving the enhanced intervention showed reduced school discipline incidents (see: https://www.pcori.org/research-results/2018/comparing-two-school-behavioral-health-programs-improve-students-academic-performance-and-mental-health (accessed on 29 July 2025)). In the following section, we describe methods focused on enhancing community engagement in this research.

## 2. Methods

### 2.1. Approach to Community Engagement

During the early stages of the project, a Stakeholder Advisory Board (SAB) was developed to continuously inform the project and sustain engagement from start to finish. Please note that the term stakeholder is still widely used in community engagement work, particularly in healthcare. The authors recognize the power of language and the historical significance of the term stakeholder (see Reed et al., 2024). The authors of this paper have backgrounds in clinical psychology, school psychology, social work, developmental psychology, and educational leadership, as well as a range of lived experiences with behavioral health challenges, parenting, and community advocacy work to support children and families. Our experiences and training informed our approaches to the analysis and interpretations in this paper. As the PSW study unfolded, community engagement beyond the bounds of the SAB informed critical adjustments to strengthen the conditions and address unexpected barriers. Each of these initiatives is described in detail below.

### 2.2. Stakeholder Advisory Board Engagement

Composition: The SAB was developed prior to the start of funding. Prospective members were identified through pre-existing connections with this study’s team members and invited by the SAB chairperson (fourth author of this paper). Some of the original SAB members participated in previous forums and focus groups. The membership criteria included the following: (a) identifying as a youth or family advocate, mental health system representative, department of education representative, researcher, or parent or staff from a participating school; (b) commitment to attend bimonthly meetings; (c) commitment to reviewing materials in anticipation of meetings and providing survey feedback after each meeting; and (d) interest and willingness to review and discuss project activities, inform family engagement roles, and play a role in disseminating study information. Once the study began, the team recognized gaps in SAB membership and responded by adding new members to represent additional groups. For instance, a school mental health clinician, principal, and parents from participating schools joined the SAB during the first year. Throughout the study, the SAB consisted of 22–26 members at a time and 31 total participants, representing state agencies, community mental health centers, youth-serving agencies, caregivers, faith community leaders, pediatricians, mental health clinicians, school principals, and researchers. Table 1 shows the distribution of the primary roles of the SAB. SAB members represented diverse races, ethnicities, genders, religions, ages/generations, educational backgrounds, and roles/professions, including those historically underrepresented in research and decision making.

Herein, we refer to these individuals—all of whom implement, receive, inform, and/or are otherwise affected by school behavioral health, which includes SAB members, study participants, and the schools and communities in which this work unfolded—as community partners. Community partner engagement was conducted with respect for autonomy, transparency, and mutual benefit. Partners were informed of the purpose of the study and their participation, their voluntary involvement, anticipated impact, incentives, and their right to withdraw at any time. All partners were provided with clear descriptions of their roles and the use of any information shared.

Training: SAB members attended a training session in the spring of 2019, before the first year of intervention implementation. The training was offered in a hybrid format to maximize attendance across sites, with most members attending in person. Led by the SAB chairperson, the training session provided information about the funder, project history, key concepts (e.g., SBH, MTSS), and features of the study (purpose, sample, conditions, and projected timeline). SAB members also learned about the elements comprising each condition and the vision for family engagement.

Structure and Format: The SAB met five times per year (every other month during the academic year). Most meetings were held via videoconference, with one meeting per year intended to be in person. The initial plan was for the full SAB to meet in person during the first, third, and fifth years of the study and for the site-based subdivisions of the SAB to meet in person during the second and fourth years of the study. Due to the COVID-19 pandemic, the SAB met virtually throughout the second, third, and fourth years, reconvening in person during the fifth year. All SAB members were invited to the bimonthly virtual meetings. Due to scheduling challenges, each one-hour meeting was also recorded so that members who were unable to attend could view the meeting, ask questions, and provide feedback after the meeting. Members were compensated for attending the meetings and watching the recordings.

Activities: Group discussions during bimonthly SAB meetings were the primary means of community engagement. Each meeting was facilitated by the SAB chairperson and included introductions (if relevant), an update on the study’s progress, the identification of current or anticipated challenges, and a discussion of members’ ideas for addressing challenges. Although discussions typically revolved around a central topic, discussions were dynamic and member-driven as opposed to being directed by the chairperson. This allowed members to respond to and build upon one another’s ideas. The chairperson worked to minimize power imbalances by setting meeting expectations and agendas, asking SAB members to contribute to agenda setting in advance, engaging in active listening, giving reflective and positive feedback during meetings, asking open-ended questions, and asking rotating members to lead specific sections of the agenda. SAB members also occasionally met in smaller groups and checked in individually with the SAB chairperson between meetings to ensure that they had multiple ways of engaging with the project and expressing their voice. The chairperson, together with a research assistant, compiled recommendations from these discussions, which were presented to the study team to determine appropriate and timely follow-up on their recommendations.

Following each meeting, SAB members completed a short survey to provide additional feedback. These surveys included a combination of ratings and open-ended questions soliciting feedback about the meeting’s structure and content (e.g., “Please share any feedback you have about the meeting in general (structure, time, length)”), members’ engagement and participation (e.g., “I was able to share any feedback I had during the meeting” on a 5-point Likert scale), and additional suggestions for solutions to specific challenges discussed at the meeting (e.g., “Please share any additional feedback you have about rewarding clinicians or other Partnership team members”). These surveys provided an alternative form of participation for members who did not have an opportunity to share or did not feel comfortable sharing their thoughts during the whole-group meeting. The surveys also funneled feedback about SAB meetings to the SAB chairperson and study team, promoting cyclical improvement of this critical study element.

Finally, the study team developed SAB newsletters, which were shared between meetings, to maintain contact, keep members up to date, and promote continued engagement with the project. Newsletters included study updates and reminders, introduced new members, provided resources on mental health, and highlighted member accomplishments external to the study to foster relational connections.

### 2.3. Participant Engagement

Although the SAB was the primary and most formal method of engaging with community partners throughout the project, study participants also interacted with the research team to provide feedback once implementation was underway. This occurred through two channels: implementation support meetings and annual focus groups. Implementation support was structured following the ECHO model (see https://projectecho.unm.edu/), as shown in Table 2. Those participating in implementation support were also invited to participate in annual focus groups to inform continuous improvement.

## 3. Results

This Results Section is organized according to PCORI engagement guidance material that details patient- and community-centered processes and guidelines to ensure engagement in research and actionable system changes, including planning the study, conducting the study, and disseminating the results. Detailed notes were kept on community partner recommendations, discussion, and follow-up action across the life cycle of this study. Illustrative examples are presented here to communicate the breadth and depth of the resulting community engagement in the PSW project.

### 3.1. Planning the Study

As reviewed above, community engagement heavily influenced the development and design of PSW. During the start-up phase of the project (prior to implementing the conditions), the SAB advised the study team on a variety of processes and decisions. The SAB significantly shaped the language used throughout the project, leading the transition from “mental health” to “wellness/well-being.” The SAB also articulated confusion surrounding the terms “patient” and “stakeholder” (both of which were adopted from PCORI), expressing a preference for “partner,” which was adopted in subsequent study materials and dissemination efforts, including this article.

The SAB was instrumental in defining some project roles (e.g., school family leader, a liaison to enhance partnerships between schools and communities) and identifying ways to integrate the school family leader into school teams. The SAB urged the study team to reconsider the training plans for school family leaders and teacher leaders (implementers of the mental health literacy curriculum). When new school family leaders and teacher leaders joined the study, the study team originally planned to send recordings of the initial training to new recruits. Based on SAB recommendations, training sessions were redesigned to be more interactive and efficient.

### 3.2. Conducting the Study

During the implementation phase, continuous community engagement became critical to sustaining the study. As challenges arose, SAB members identified possible solutions and offered considerations for how participants might be impacted. During the first year of the study, recruitment was slow, resulting in lower-than-anticipated numbers of students receiving SBH services and enrolling in the study. Calling upon their personal and professional experience, SAB members identified problematic jargon and recommended reducing the paperwork burden and removing requirements for in-person meetings to complete the consent process. In response, the study team simplified the consent process and materials, developed a virtual consent procedure, shifted from in-person to electronic modalities (phone, videoconferencing, and email/text) for communication and data collection, and created a whiteboard video explaining the study in plain language, which was uploaded to YouTube and included in the online consent process for caregivers.

Given the feedback from clinicians that some families were wary of research, SAB members brainstormed alternative language to allay concerns. To expand reach and enhance recruitment, the SAB suggested enhancing the marketing of SBH services in school buildings, recommending outreach through school events (e.g., back-to-school nights and sporting events) and more group-based (vs. individual) services. In response, study clinicians offered more group-based services, inviting teachers to make direct referrals for students showing signs of distress. During implementation support meetings, clinicians engaged in ongoing discussion about recruitment and enrollment processes, sharing strategies that worked in their buildings.

Based on feedback received from SBH clinicians and school family leaders, the SAB recommended that the study team supply school family leaders with helpful information to share with their school and caregivers in their school community in order to assist in content creation. The study team shared materials created by the National Institute of Mental Health and developed new content-specific one-pagers. In response to confusion surrounding the school family leader role, the SAB developed a menu of mental health promotion activities that school family leaders could engage in, including contributing to school newsletters and caregiver emails, hosting informational tables at school events, and posting on school websites or social media pages. Many of these suggestions were implemented at school sites, although they were adopted as requirements for the school family leader role. The SAB also advocated expanding the number of hours relative to which school family leaders were compensated each year, given the evolving demands of the position.

Community partners provided valuable feedback used to adapt and implement the mental health literacy curriculum during the pandemic. SAB members identified concerns with developmental appropriateness, recommending reducing brain-based content to improve engagement with the material. In response, the mental health literacy curriculum was adapted to improve both the developmental and cultural relevance of the content and materials. They suggested emailing parents an overview of the curriculum and sharing the curriculum with parents during parents’ night to increase visibility and understanding. In response to hesitations expressed by teachers, the SAB recommended exposing all teachers to the material at the beginning of the school year to build familiarity, collecting student and teacher feedback to inform changes, providing teams with greater clarity on which content had been covered each school year to avoid redundancy, and augmenting implementation support. The study team followed these recommendations and invited the developers of the mental health literacy curriculum to implementation support meetings, which teachers reported to be helpful.

SAB input also informed the approach to cultural responsiveness and equity. For instance, SAB members recommended providing additional training to teachers on cultural responsivity, which occurred between the first and second years of implementation. The SAB also suggested that school teams develop plans to share bias-related information with their school community. In response, the study team developed a template for a “Mental Health Moment” flyer that could be adapted and shared with teachers and other school staff to communicate bias-related information alongside mental health content. The SAB reviewed and provided feedback on the project’s cultural responsiveness and equity materials, identifying perceived strengths and weaknesses. Strengths were retained, and weaknesses were discussed by the study team for potential improvement. The SAB also highlighted the need for increased clarity when using terms like “bias,” noting the importance for partners to have a common language for discussing the project and mental health in general.

Finally, feedback loops connecting students and clinicians with the study team informed approaches to overcoming additional barriers throughout the project. Through implementation support meetings, clinicians shared that students frequently expressed that surveys took too much time to complete. With guidance from the study team, clinicians sought additional student input on which specific measures were overly arduous and relayed that information to the study team to determine possible solutions. In response, the study team met with the measure developer and a statistician to determine which items could be removed without impacting psychometric validity. As a result, the student surveys were shortened, alleviating the burden on students and clinicians. Clinicians also provided feedback on the implementation of study elements, which informed changes to subsequent trainings and implementation plans. For instance, they expressed frustration with managing teachers’ monthly data reports, noting that this role placed them in a dual relationship as both a colleague and manager. The study team eliminated monthly reports for teachers and allowed clinicians to gather data in a more informal, collaborative way, which was met with an overwhelmingly positive response by the clinicians.

In general, SAB members advocated for the well-being of study participants, including students, families, clinicians, and educators, reminding the study team to prioritize the community’s needs in decision making. This ultimately informed the decision not to extend the study for an additional year (related to disruptions from the pandemic), as SAB communicated that clinicians and educators did not perceive the added benefit to be worth the extra workload. Based on the SAB recommendation, continued education credits were also awarded to individuals implementing study elements to support their additional time and effort.

### 3.3. Disseminating Results

**Figure 2:**
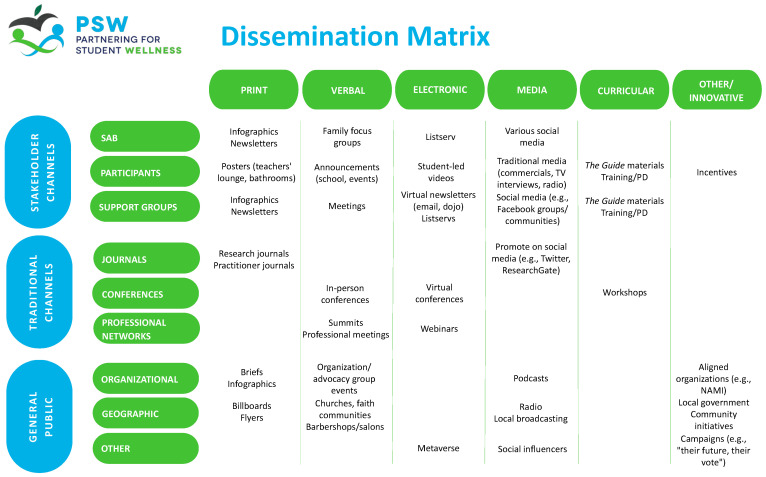
Dissemination matrix completed by the SAB.

Conversations with SAB surrounding dissemination began halfway through the project, and planning became more focused during the final year of implementation. During this phase, the SAB was instrumental in defining the dissemination process and approach, identifying key audiences, brainstorming strategies for communicating effectively with diverse audiences, and developing a variety of dissemination products. To help orient the SAB to the concept of dissemination, the SAB chairperson developed two tools: a dissemination model and a dissemination matrix. The dissemination model was based on a multilevel ecological framework, depicting communities with varying degrees of proximity to the project. The dissemination matrix (Figure 2) crossed dissemination modalities (e.g., print, verbal, electronic, etc.) with dissemination channels (e.g., stakeholder channels, traditional channels, and channels for reaching the public) to portray the need to consider both how and to whom study information should be disseminated. SAB members identified points of confusion and then provided feedback on changes to the language and content of the tools, which were modified in response.

Initially, SAB members displayed hesitance and reported not feeling equipped to provide guidance on dissemination. In response, the study team brought the first author on board to dedicate time exclusively to guiding dissemination discussions, collecting and analyzing feedback, facilitating activities, and supporting SAB members in navigating the dissemination process with ongoing training and consultation during and between SAB meetings. SAB members significantly shaped the dissemination process by defining objectives, offering considerations, and specifying products and strategies. The SAB identified key audiences, prioritized findings for dissemination, generated strategies for communicating specific findings to different audiences, and provided feedback on products to optimize them for the intended audience. For example, SAB members ranked different study findings according to their perceived importance and interest, reported the different means through which they receive information pertaining to their professional roles and their daily lives, and indicated their preferences for methods of receiving and sharing information. At an in-person meeting, the SAB participated in an interactive activity in which they collaboratively populated the cells in the dissemination matrix. This completed matrix continues to be reviewed regularly to guide the development of dissemination products and the selection of strategies.

The study team has partnered with SAB members and study participants in the development of dissemination products. Participants, including teachers, clinicians, and school administrators, have collaborated with study team members to present findings at multiple conferences. Participants have also been involved in developing and reviewing consumer products (e.g., informational booklets, curricular materials, training materials), which has been critical to optimizing their utility to end users. Recently, a website was developed and refined through multiple open meetings with community members to ensure that the materials and formatting were optimized for a community audience (see www.partneringforstudentwellness.com). These efforts are ongoing, with the study team collaborating with the SAB and participants to explore new opportunities for community-engaged dissemination.

## 4. Discussion

As mentioned, our approach to community engagement developed over 13 years, including the development of a state and then regional community of practice (see Cashman et al., 2014), obtaining an Engagement Award from PCORI, with findings from this award contributing directly to a PCORI-funded comparative effectiveness trial. This progressive and deepening community engagement strategy enabled rich, bidirectional communication, fostering an environment where study team members and community partners contributed to mutual learning. The ongoing dialogue and sharing of diverse perspectives and experiences among participants enriched the research experience and were critical to intervention selection, design, and implementation in the comparative effectiveness trial. Collaborating with community members at each stage of the research process enhanced the relevance of interventions, ensuring that intervention strategies were aligned closely with community needs and priorities, materials and procedures were adapted to fit the local context, and adjustments were continually made to sustain intervention implementation as challenges arose.

The PCORI engagement principles (reciprocal relationships, co-learning, partnerships, transparency, honesty, and trust; PCORI, 2014) informed our approach to community engagement across both projects. Recently, PCORI expounded upon these engagement principles in their publication of six foundational expectations for meaningful, effective, and sustainable community engagement (PCORI, 2024). Below, we reflect on the extent to which we met these expectations and identify opportunities for improvement in future community-engaged research.

### 4.1. Diversity and Representation

PCORI stipulates that partners reflect the diversity of the individuals and communities affected by the research (PCORI, 2024). Our SAB represented the interdisciplinary fields characterizing SBH and the demographics of the schools and communities participating in the research. Members were diverse in race, ethnicity, gender, religion, age/generation, education, and role/profession and reflected multiple communities that have been historically excluded from research. However, some aspects of identity, such as ability, socioeconomic status, national origin, and sexual orientation (see Hays’ ADDRESSING framework; Hays, 1996), were not deliberately sought in the process of identifying key community partners. When individuals (typically, advisory board members) vocalized a need for additional communities to be represented, new individuals were invited to the SAB. This resulted in greater diversity of school professionals and community-based youth-serving organizations. Structuring engagement activities required dexterous steering to ensure inclusive yet effective participation. For example, when some voices monopolized the discussion, the SAB chair redirected the discussion by refocusing on the question at hand and specifically inviting other participation and offering one-on-one contacts following the meetings to ensure that everyone had the opportunity to express their views.

### 4.2. Early and Ongoing Engagement

PCORI expects meaningful community engagement throughout the entire lifespan of a project, including planning the study, conducting the research, and disseminating findings (PCORI, 2024). The large scope and longitudinal nature of the final comparative effectiveness trial required managing fluctuating engagement over time, necessitating continuous onboarding efforts to integrate new members into an established group. Logistical issues, such as Zoom fatigue and scheduling conflicts, also complicated engagement. In response, we diversified engagement methods, including alternatives like recorded sessions, Google Forms for asynchronous feedback, one-on-one calls, and regular newsletters to maintain connection. While not every attempt was successful, maintaining an open dialogue with partners and cultivating honesty and trust through the transparent and reciprocal sharing of ideas fostered continuous problem-solving. By recognizing our community partners’ strengths, respecting their preferences, and offering a variety of opportunities for engagement, we were able to sustain high levels of community engagement throughout each phase of the project.

### 4.3. Dedicated Funds for Engagement and Partner Compensation

PCORI requires that community partners be compensated for their time and efforts in ways that align with their preferences (PCORI, 2024). Budgeting for community engagement on the frontend is essential to achieving active, meaningful, and sustained engagement that benefits researchers and community partners (Leisey et al., 2022). This establishes community engagement as a priority and ensures that adequate resources are allocated to fostering and monitoring engagement. Our SAB members and other participants were compensated for every meeting attended (or watched, if they could not attend) and time spent on efforts beyond meetings (such as developing, reviewing, and refining materials). Engagement funds allowed community partners to travel to connect with one another in person at an annual conference. Dedicated funds also enabled the assignment of personnel to engagement activities, such as chairing the SAB and coordinating community-engaged dissemination activities.

### 4.4. Build Capacity to Work as a Team

Identifying community partners’ skills, strengths, and engagement barriers and using that information to provide appropriate training and support is critical to successful engagement (PCORI, 2024). Early in the project, after the initial group of SAB members was recruited, we held a hybrid training session that offered detailed information on the study and the engagement initiative (SAB) and a vision for their involvement. Although we appreciated the diversity in the lived experience of our SAB members and recognized their differing perspectives and skillsets, we did not formally assess partners’ skills, strengths, or engagement barriers. In retrospect, this placed the responsibility of advocacy on SAB members, who often did vocalize concerns that they lacked the required skills to follow through on a recommendation or encountered a life circumstance that interfered with engagement. We aimed to meet our partners where they were at each step of the project, providing additional information and support in response to their feedback through newsletters, handouts, videos, and conversations.

These efforts were generally well-received and helped our partners participate to the full extent of their choice. That said, a more proactive approach to identifying partner strengths, skills, and barriers may facilitate greater tailoring and prevent some of the challenges that we experienced. For instance, we encountered hesitancy in some community partners when it came to disseminating findings. After five years of collaborative work with our SAB, we assumed that our community partners had the same understanding, motivation, and eagerness to disseminate learnings as members of the study team. We quickly learned that many partners lacked confidence in their ability to contribute to dissemination, and we changed our approach accordingly. This involved assessing partners’ preferences and comfort with dissemination, tailoring our discussions and activities to our partners’ interests and pace, and scaffolding dissemination activities through modeling and examples. This experience highlights the importance of attending to capacity-building to support meaningful participation of partners across all research stages (Isler & Corbie-Smith, 2012). This might include improving training (or providing ongoing training), preparing partners for meetings by providing materials in advance, soliciting feedback, evaluating engagement initiatives, and intentionally fostering the development of self-confidence in their skills, such as through peer support (De Weger et al., 2018; Matthews et al., 2018).

### 4.5. Meaningful Inclusion of Partners in Decision Making

Ideally, community partners are involved in decision making throughout each phase of the project (PCORI, 2024). This includes considering partner perspectives when making decisions, addressing power imbalances, being transparent about decision making, and fostering shared leadership and responsibility (PCORI, 2024). Partner perspectives influenced decisions, both big and small, at each step of the project. We continuously solicited ideas and opinions on everything, from naming this study’s conditions and recruiting additional SAB members to intervention materials, recruitment strategies, support for teachers and clinicians, dissemination methods, and, ultimately, whether to extend the project.

One recurrent tension we experienced was balancing the need for structure and control in a comparative effectiveness trial with the flexibility required to adapt to community feedback (Buchanan et al., 2007). Relatedly, some points of community feedback were infeasible given budgetary or staffing constraints. Thus, some valuable insights and recommendations that may well have improved implementation and/or impact were not acted upon. We kept detailed notes on community partner recommendations and promoted transparency and shared responsibility by following up with our partners to share which recommendations were acted upon. At times, our community partners expressed confusion or disappointment over recommendations that were not followed. We encouraged these expressions, which we interpreted as a positive indicator of engagement and participatory value. We aimed to listen actively and humbly and to convey our partners’ perspectives to the study team so they could be weighed in decision making.

### 4.6. Ongoing Review and Assessment of Engagement

PCORI (2024) recommends that teams adopt an iterative approach to evaluating engagement to inform adjustments to the engagement strategy. We formatively evaluated engagement throughout the project through brief surveys following each SAB meeting and invited informal feedback through individual and group discussions. Ratings of engagement were generally high, and partners occasionally provided suggestions for improvement (e.g., offering longer meetings, changing the meeting time, providing more information upfront), some of which were incorporated. In hindsight, it may have been helpful to have more detailed formative evaluation data to better understand engagement strengths and weaknesses and tailor the approach accordingly. Indeed, this is recommended in the literature on engagement and noted as instrumental to building partners’ capacity for engagement (Matthews et al., 2018).

### 4.7. Socio-Historical Context

This study coincided with the COVID-19 pandemic, which disrupted schools, reshuffled priorities, and threatened both the project’s completion and the delivery of support to students in participating schools. However, strong community engagement fostered resilience, enabling the study to continue when many others faltered (Audisio et al., 2022). Community members’ insights facilitated rapid pivoting and responsive decision making, showcasing the value of community engagement in creating adaptable, contextually relevant solutions to unprecedented challenges. Crucially, this study’s success was rooted in longstanding relationships with the SAB, which were nurtured over many years and strengthened through in-person training and frequent contact before and during the pandemic. This contact nurtured shared accountability for the project, laying the foundation for a successful transition to virtual engagement with minimal turnover. Unexpectedly, the virtual environment also augmented engagement by providing new opportunities for cross-site collaboration, facilitating a greater exchange of ideas, and producing more creative and workable solutions than may have otherwise emerged.

### 4.8. Levels of Engagement

Key et al. (2019) defined a continuum of community engagement in research, ranging from no community involvement to community-driven/community-led research. The activities we (researchers and community partners) engaged in over the course of this project represent multiple levels of engagement. Rather than characterizing the project as a whole, the community-engaged research continuum is helpful for understanding how community engagement fluctuated over time and across partners/relationships at a given time. In reality, community engagement was dynamic, and different aspects of the project were simultaneously characterized by multiple levels of community engagement. For instance, from the perspective of some community partners who partook in the forums and focus groups that informed the comparative effectiveness trial but were not involved in specific research decisions, the project might seem “community-informed.” Other (fewer) community partners were intimately involved with the trial during the planning stage and also implemented interventions in the schools and served on the SAB and the study team, playing a greater role in decision making. From their perspective, this project may be perceived as akin to community-based participatory research (see Collins et al., 2018). Some relationships and activities also exemplified community consultation, community participation, and community initiation. The principles and ethics of participatory research heavily informed our approach to community engagement; however, we had to make compromises related to the research design and scale of the project. Ultimately, we found that combining clear principles with a pragmatic approach yielded levels of engagement that defied definition but were no less meaningful or impactful. Regardless of the level of engagement, we illustrate that both contextual factors (history, trust, relationship building, respect, and transparency) and equity factors (power and control, decision making, influence, mutual benefit, ownership, responsibility, and resource sharing) articulated by Key et al. (2019) and PCORI (2024) were foundational to the sustainability of our community engagement efforts.

### 4.9. Future Directions

As researchers increasingly partner with communities and policymakers in research intended to bring about positive change through ongoing project action cycles, several future directions can be informative in sustaining relationships. First, sharing information at the start of a project about the ways in which project decisions will be made and proactively providing the rationale behind decisions throughout the project can enhance transparency, potentially fostering greater trust and helping to balance power. Second, openly communicating about the engagement budget and inviting discussions about the use of these funds can increase transparency and may reveal innovative ways community partners can utilize funds to further study aims. Third, the creation and implementation of activities to identify partner strengths, skills, and barriers near project start-up and frequent targeted formative evaluation can support partner engagement and contributions. Dozens of tools—with varying degrees of comprehensiveness, partner engagement in tool design, psychometric soundness, and usability—have been developed and used to evaluate health research partnership outcomes (Mrklas et al., 2023). However, the extent to which these tools have been used, or are appropriate to use, for formative evaluation is unclear. Ongoing research and evaluation are needed to identify the extent to which the above goals are achieved, with an eye toward community impact and sustainable and equitable partnering outcomes. Systematic investigation of representation and engagement experiences of key partners across multiple projects could further guide the practice of community engagement in school behavioral health. Finally, it is imperative that funders, universities, and relevant governmental agencies adopt and communicate structures, expectations, and tools that enable and support ongoing and meaningful community engagement. Supportive policy and infrastructure, such as that provided by PCORI, underpin the successful translation of research ideals into actionable strategies that can build and sustain impactful research–community partnerships.

## Figures and Tables

**Table 1:** Stakeholder advisory board member representation.

Primary Representation	Number of Representatives
Community Faith Leader	1
Education Agency	1
Family Advocate	4
Family Organization	3
Health Foundation Organization	1
Medicaid Agency	1
Mental Health Agency	5
Mental Health Organization	1
Parent	2
Principal	1
Educator	2
Rural Student Organization	1
School District	2
School-based Clinician	2
University Pediatric Mental Health	1
University Researcher	2
Youth Advocate	1
TOTAL	31

**Table 2:** Implementation support following the ECHO model.

Role	Activities	Frequency	Feedback and Evaluation
School mental health clinicians	Didactic instruction on clinical skills Case presentation and peer discussion Applied activities to practice skills	Every other week	Discussion Feedback form with ratings and open-ended responses about topic, takeaways, and utility
Implementers of the mental health literacy curriculum	Training Expert consultation Discussion of implementation experiences or community outreach (“cases”) Peer-to-peer support	Monthly	Discussion Feedback form with key takeaways and an opportunity for questions
School family leaders (SFLs)	Guidance on SFL activities School connection sharing (“cases”) Two formal expert didactics	Monthly	Discussion
Experts/study team	Hosted implementation support meetings	As indicated above	Compiled and used formative feedback on evaluations

## Data Availability

No dataset will be made available to the public.

## Abbreviations

The following abbreviations are used in this manuscript:

SBH	School Behavioral Health
PCORI	Patient-Centered Outcomes Research Institute
MTSS	Multi-Tiered Systems of Support
CBPR	Community-Based Participatory Research
SCSBHC	South Carolina School Behavioral Health Community
EBP	Evidence-Based Practices
SSBHC	Southeastern School Behavioral Health Community
PSW	Partnering for Student Wellness
SAB	Stakeholder Advisory Board
